# UV and Visible Light-Driven Production of Hydroxyl Radicals by Reduced Forms of N, F, and P Codoped Titanium Dioxide

**DOI:** 10.3390/molecules24112147

**Published:** 2019-06-06

**Authors:** A. M. Abdullah, Miguel Á. Gracia-Pinilla, Suresh C. Pillai, Kevin O’Shea

**Affiliations:** 1 Department of Chemistry & Biochemistry, Florida International University, Miami, FL 33199, USA; am.abdullah@fiu.edu; 2 Facultad de Ciencias Físico Matemáticas, Universidad Autónoma de Nuevo León, Av. Universidad s/n, Cd. Universitaria, San Nicolás de los Garza, Nuevo León 66455, Mexico; miguel.graciapl@uanl.edu.mx; 3 Nanotechnology and Bio-engineering Research Group, Department of Environmental Science, Institute of Technology, Sligo F91 YW50, Ireland; pillai.suresh@itsligo.ie

**Keywords:** visible light activated titanium dioxide, visible light activated TiO_2_, hydroxyl radical, hydroxyl radical quantification, photocatalytic activity, coumarin

## Abstract

The photocatalytic activities of reduced titanium dioxide (TiO_2_) materials have been investigated by measuring their ability to produce hydroxyl radicals under UV and visible light irradiation. Degussa P25 TiO_2_ was doped with nitrogen (N), fluorine (F), and/or phosphorus (P) and then subjected to surface modification employing a thermo-physicochemical process in the presence of reducing agent sodium borohydride (NaBH_4_). The reduced TiO_2_ materials were characterized by a number of X-ray, spectroscopic and imaging methods. Surface doping of TiO_2_ was employed to modulate the band gap energies into the visible wavelength region for better overlap with the solar spectrum. Hydroxyl radical generation, central to TiO_2_ photocatalytic water purification applications, was quantitated using coumarin as a trap under UV and visible light irradiation of the reduced TiO_2_ materials. At 350 nm irradiation, the yield of hydroxyl radicals generated by the reduced forms of TiO_2_ was nearly 90% of hydroxyl radicals generated by the Degussa P25 TiO_2_. Hydroxyl radical generation by these reduced forms of TiO_2_ was also observed under visible light irradiation (419 and 450 nm). These results demonstrated that simple surface modification of doped TiO_2_ can lead to visible light activity, which is important for more economical solar-driven applications of TiO_2_ photocatalysis.

## 1. Introduction

While titanium dioxide (TiO_2_) has attracted remarkable attention as a photocatalyst over the past few decades for the treatment of wastewater and the degradation of hazardous chemicals, significant industrial applications and commercial benefits have yet to be realized [1,2,3]. TiO_2_ photocatalysis requires absorption of a photon, typically in the UV region (λ < 400 nm), to generate an electron (e^−^_CB_)/hole(h^+^_VB_) pair, Equation (1), which can initiate degradation through the production of reactive oxygen species (ROS) as outlined below in Equations (1)–(5). The recombination of the electron/hole pair is often a dominant process which eliminates the potential for the formation of ROS. In competition with recombination, the electron/hole pairs can migrate to the surface of TiO_2_ and form ROS in the presence of water and oxygen [4,5,6,7]. While direct oxidation and reduction of target substrates by electron/hole pair can also be envisioned, such direct transformations are generally not competitive in aqueous solution. The primary processes leading to degradation of target pollutants usually involve the formation of ROS, hydroxyl radicals (•OH), Equations (2) and (5), superoxide anion radicals (O_2_^•−^), Equation (3), and hydrogen peroxide (H_2_O_2_), Equation (4). Although a number of ROS are generated in a TiO_2_ photo-oxidation process and can lead to the mineralization of organic pollutants, hydroxyl radical (•OH) is considered the primary species leading to degradation [8,9,10].

TiO_2_ + *hv* → h^+^_VB_ + e^−^_CB_ (1)

H_2_O + h^+^_VB_ → H^+^ + •OH (2)

O_2_ + e^−^_CB_ → O_2_^•−^ (3)

2H_2_O + 2O_2_^•−^ → H_2_O_2_ + 2HO• + O_2_ (4)

H_2_O_2_ + e^−^_CB_ → 2HO• (5)

**Figure 1:**
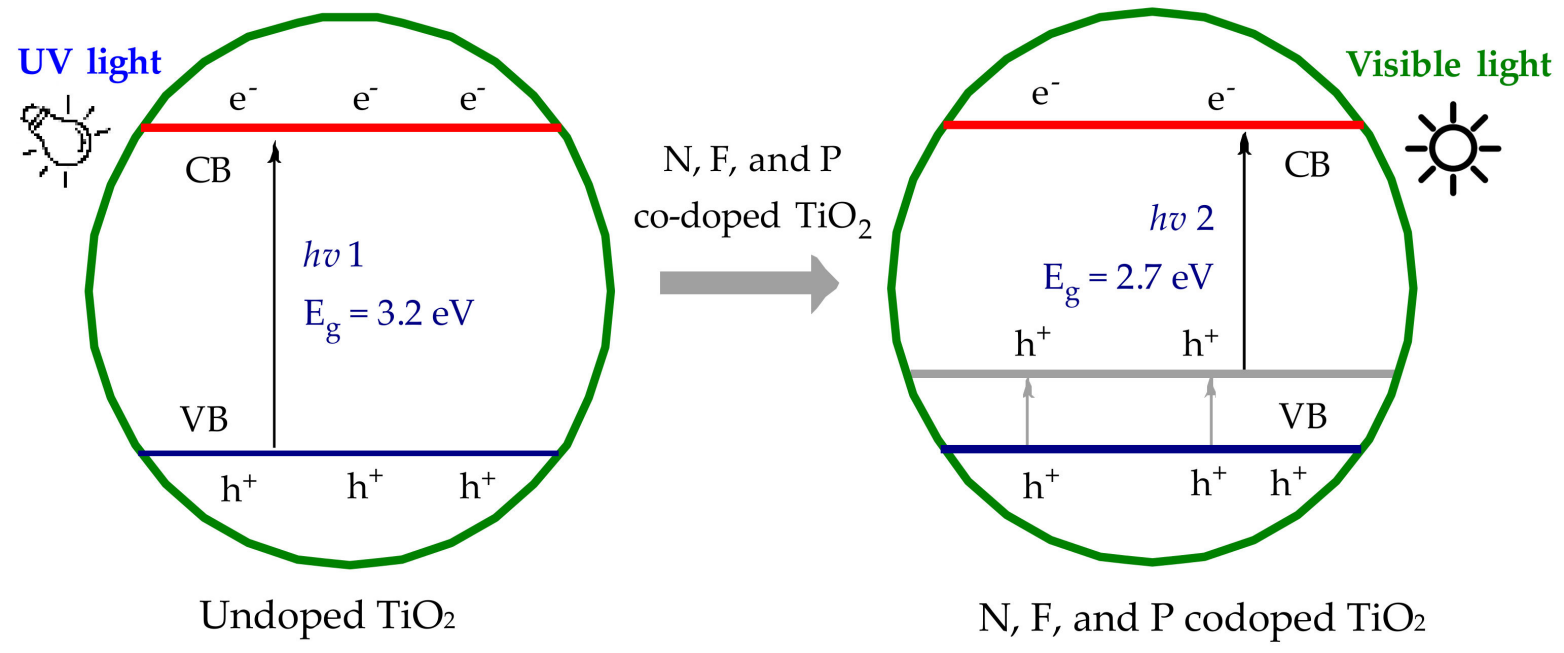
Schematic band gap energy levels of doped and undoped TiO_2_; E_g_ stands for the band energy gap between the valance band (VB) and conduction band (CB).

TiO_2_ is readily available, inexpensive, and chemically stable; however, there are two major drawbacks to TiO_2_ photocatalysis [3,11]. Firstly, the photo-excitation of unmodified TiO_2_ requires UV light. Secondly, the quantum yield for photochemical production of ROS is low due to the rapid recombination of electron/hole pairs [7,12]. The solar spectrum is predominately in the visible region, with only ~5% of the total irradiation in the UV region. A major challenge in TiO_2_ photocatalysis is to utilize the visible region of the solar spectrum. Extension of the TiO_2_ light absorption profile into the visible light region offers significantly improved economic viability compared to those requiring UV activation. A number of techniques including nonmetal doping with nitrogen (N), sulfur (S), carbon (C), fluorine (F), iodine (I), or boron (B), nonmetal codoping, metal deposition, dye sensitization, semiconductor coupling, and enriching the oxygen content of TiO_2_ have led to a variety of visible light activated (VLA) TiO_2_ materials over the last decade [1,3,13,14,15,16,17,18,19,20]. A number of metal doping materials has been previously explored; however, metal doping often produces undesirable metal titanates at high processing temperatures [21,22,23,24]. Nonmetal doping or codoping into the TiO_2_ modifies the valence band level of TiO_2_, reducing the effective band gap energy, as shown in Figure 1, and modulating the light absorption into the visible light region [25,26,27,28,29,30]. Although the incorporation of dopant elements can result in the advantage of narrowing band gap energies, dopants can influence charge carrier recombination processes, and the limited solubility of dopants into the bulk materials as well as thermal instability have limited photocatalytic applications of doped TiO_2_ materials [31,32,33,34,35]. 

Simple reduction of Ti within the TiO_2_ matrix leads to stable colored TiO_2_ with visible light absorbing properties, and these materials have attracted extensive attention due to their enhanced photocatalytic activity [36,37,38,39]. Notably, black TiO_2_ prepared from the hydrogenation of TiO_2_ nanocrystals at high-pressure exhibits relatively low band gap energy and enhanced photocatalytic activity for water splitting and dye degradation [36]. A number of approaches, including high pressure, high temperature, plasma-assisted hydrogenation, and high-temperature aluminum vapor reduction, have been explored for preparation of VLA TiO_2_ materials [37,38,39,40]. High-pressure and/or high-temperature protocols are often experimentally challenging and/or expensive. Recently, Tan et al. developed a fast and simple chemical reduction method for the production of colored TiO_2_ through a controllable solid-state reaction of NaBH_4_ and crystalline TiO_2_ at moderate temperature (< 300 °C) [41]. The resulting reduced TiO_2_ material possessed a narrow band gap with a crystalline core/amorphous shell structure (TiO_2_@TiO_2 − x_) containing oxygen vacancies at the surface of TiO_2_ promoting the separation of charge carriers and leading to improved VLA photocatalytic performance. Herein, we synthesized reduced codoped P25 TiO_2_ through thermal treatment in the presence of NaBH_4_ in an attempt to extend the TiO_2_ photocatalytic function into the visible light region. 

Although reduction and codoping of TiO_2_ can narrow band gap energies appropriate for the absorption of visible light, enhanced visible light photocatalytic activity is not ensured [3]. The photocatalytic activity is generally associated with a redox reaction initiated by the photogenerated charge carriers. Assessment and comparisons of the photocatalytic activity of different TiO_2_ materials are challenging due to the variation of experimental conditions, equipment design, flux intensity, loading, band gap, surface area, and crystal lattice and composition. While the application of TiO_2_ photocatalysis for remediation of pollutants and toxins in aqueous media has been studied extensively, assessing the specific effectiveness of the photocatalyst is still not standardized [42,43,44,45,46,47]. Since •OH is generally the primary species initiating the degradation of target pollutants during TiO_2_ photocatalysis, monitoring •OH production during TiO_2_ photocatalysis is an effective way to evaluate TiO_2_ photocatalytic activity. Noteworthy, Xiang et al. reported an OH-index parameter, as expressed in Equation (6), to assess the activity of a photocatalyst [47].

OH-index = (r/r_0_) × 100 (6)

where r and r_0_ are the formation rate of •OH on the irradiated photocatalyst and reference photocatalyst P25 TiO_2_, respectively. P25 TiO_2_ was taken as the reference standard for the production of •OH under UV irradiation as one of the most extensively investigated and most active commercial photocatalysts [46].

**Scheme 1:**
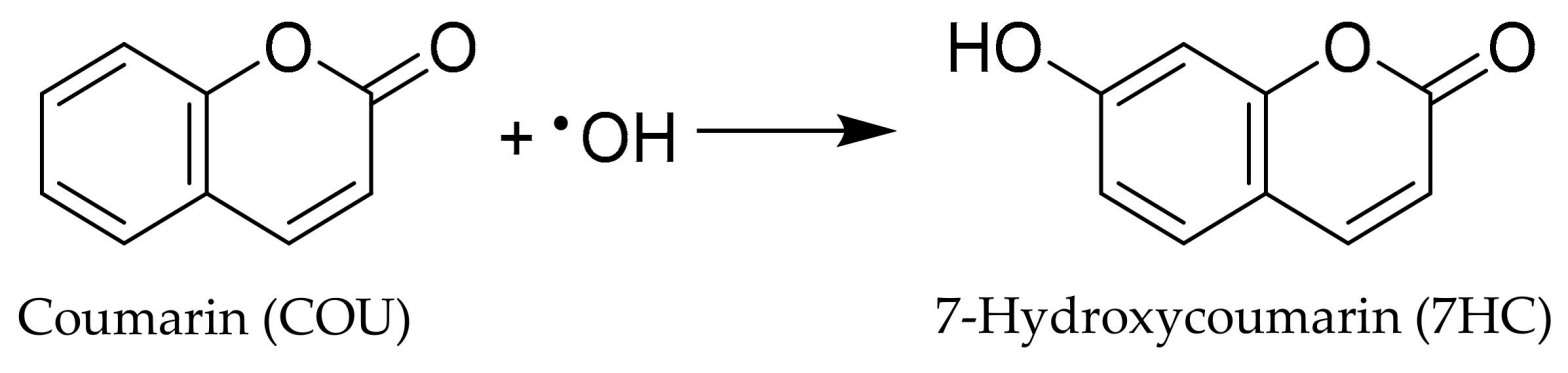
The reaction of COU and •OH for highly florescent 7HC production.

Direct measurement of •OH is difficult because its short wavelength absorbance (<230 nm) makes it difficult to monitor and its high reactivity results in an extremely short lifetime (often 10^−9^ s) [10]. Given these limitations, indirect methods have been established to trap hydroxyl radicals with the formation of stable hydroxyl radical adducts. Among hydroxyl radical traps, coumarin (COU) forms a hydroxyl radical adduct which can be readily quantitated using fluorescence spectroscopy. We have employed coumarin which yields 7-hydroxycoumarin (7HC) upon trapping of hydroxyl radical, as shown in Scheme 1. The concentration of 7HC measured by fluorescence is proportional to the production of hydroxyl radicals by TiO_2_ materials under UV and visible light [48]. 

One of the purposes of this study was to develop visible light active forms of TiO_2_ through a simple reduction process. We prepared the VLA TiO_2_ materials using a controllable solid state thermal physicochemical process. Detailed physical and chemical characterization of the prepared materials, along with their optical properties, were carried out by a number of techniques including X-ray powder diffraction (XRD), high-resolution transmission electron microscope (HRTEM), energy dispersive X-ray fluorescence (EDXRF), ultraviolet-visible (UV-Vis) spectroscopy, and Raman spectroscopy. Hydroxyl radical production is reported as a measure of the viability of these TiO_2_ materials for the destruction of environmental contaminants in potential water treatment applications. The study also compares the relative photocatalytic efficiency of prepared TiO_2_ materials using OH-index. Our results provide insight into the development of visible light-activated photocatalysts for the destruction of organic pollutants in water.

## 2. Results and Discussions

**Figure 2:**
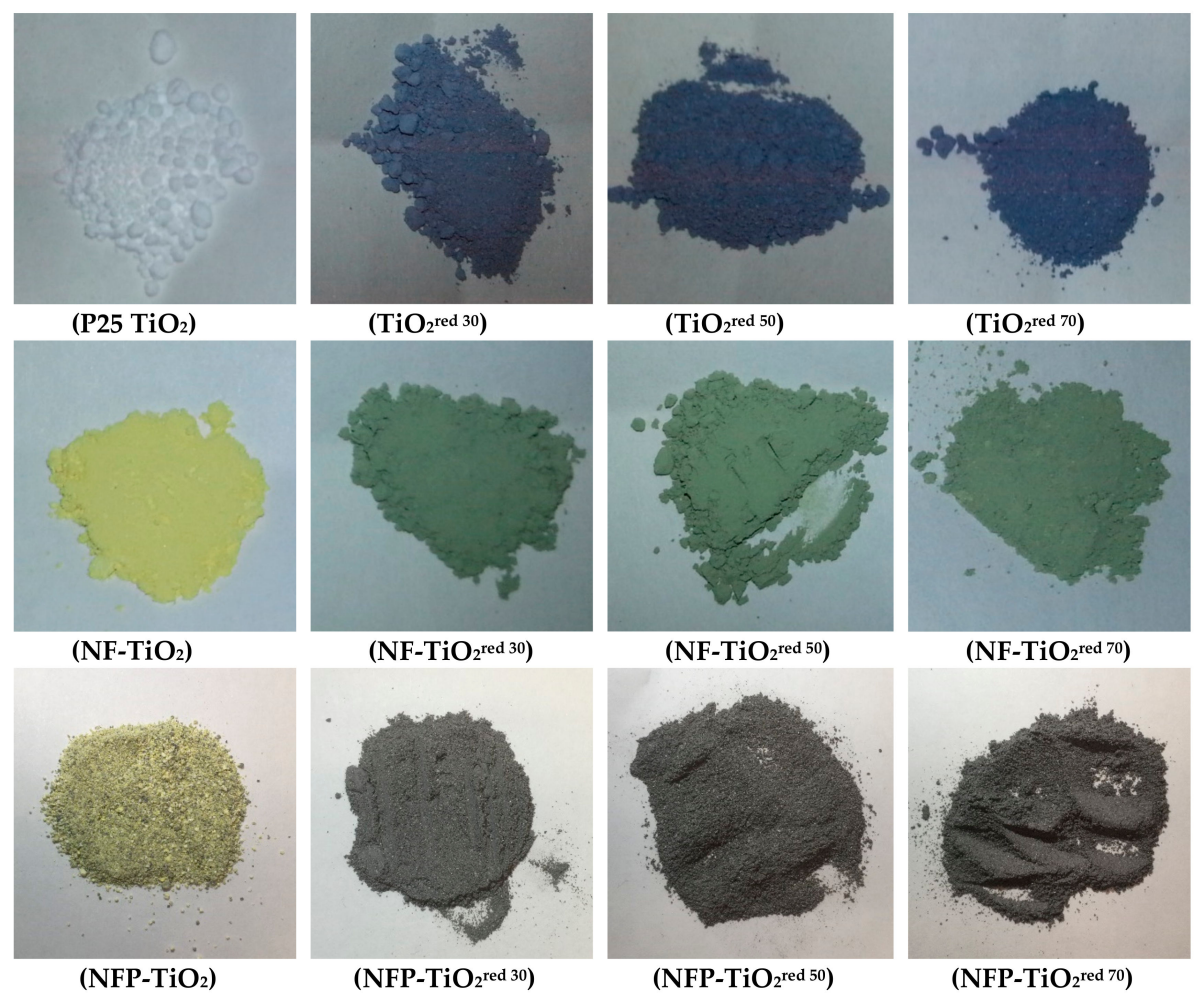
Color photographs of reduced TiO_2_ synthesized by thermal treatment with NaBH_4_.

A series of reduced TiO_2_ materials (identified as TiO_2_^red^) for P25 TiO_2_ and N, F, and/or P codoped TiO_2_ (NF-TiO_2_ and NFP-TiO_2_) were synthesized by thermal treatment with NaBH_4_ for 30, 50, and 70 min following the reported method [41] as detailed in the experimental section. The specific conditions of thermal treatment used in the preparation of reduced TiO_2_ materials are tabulated in Table 1, and the optical reflectance of representative samples are shown in Figure 2. 

### 2.1. Phase Structure Analysis

**Figure 3:**
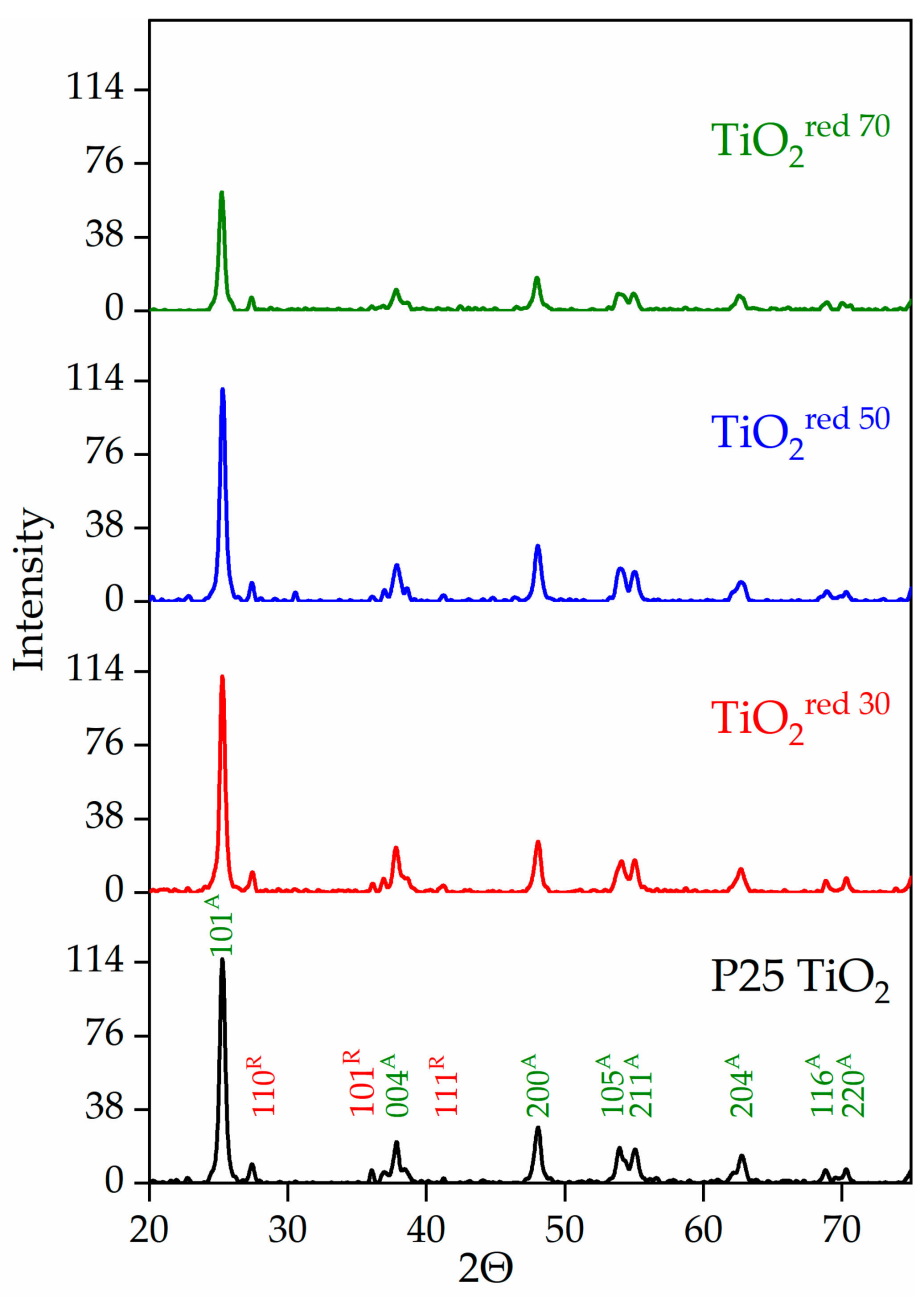
Comparison of XRD patterns among P25 TiO_2_ and reduced forms of P25 TiO_2_ materials (green and red Millar indices represent the anatase and rutile phases, respectively).

The performance of a photocatalyst is often dependent on the many factors, including the crystal structure, surface area, and composition [18,22,49]. TiO_2_ can exist as three different forms; anatase, rutile, and brookite [21,49,50,51,52]. Anatase is considered the most photocatalytically active crystal form. The photocatalytic activity of TiO_2_ can vary dramatically based on its lattice structure, and thus, X-ray powder diffraction (XRD) patterns were used to analyze the crystalline properties of synthesized TiO_2_ samples. The XRD data of P25 TiO_2_ and reduced TiO_2_ are provided in Figure 3. All observed peaks in the XRD patterns of the reference P25 TiO_2_ and the reduced TiO_2_^red^ were indexed to anatase and rutile phases. The intensity of XRD peaks for TiO_2_^red 70^ associated with the anatase phase, however, decreased, indicating lower crystallization due to surface modification at longer treatment times without a significant change of crystallinity. Anatase (tetragonal structure, a and b) 3.78 Å; c) 9.50 Å) and rutile (tetragonal structure, a and b) 4.58 Å; c) 2.95 Å) are the two major forms of crystalline TiO_2_. Titanium oxide can also exist in another thermodynamically unstable form called brookite (rhombohedral structure, a) 5.43 Å; b) 9.16 Å; c) 5.13 Å). The brookite phase was not detected in any of the samples in this study. Previous experiments showed that anatase is kinetically more stable than rutile while rutile is reported to be more thermodynamically stable than anatase at normal atmospheric pressure and temperature [11,51]. It has been found that a crystalline mixture of the two different phases of titanium dioxide, such as anatase and rutile is more photoactive than 100% anatase [51,52,53]. 

### 2.2. Microstructural and Morphological Analysis

**Figure 4:**
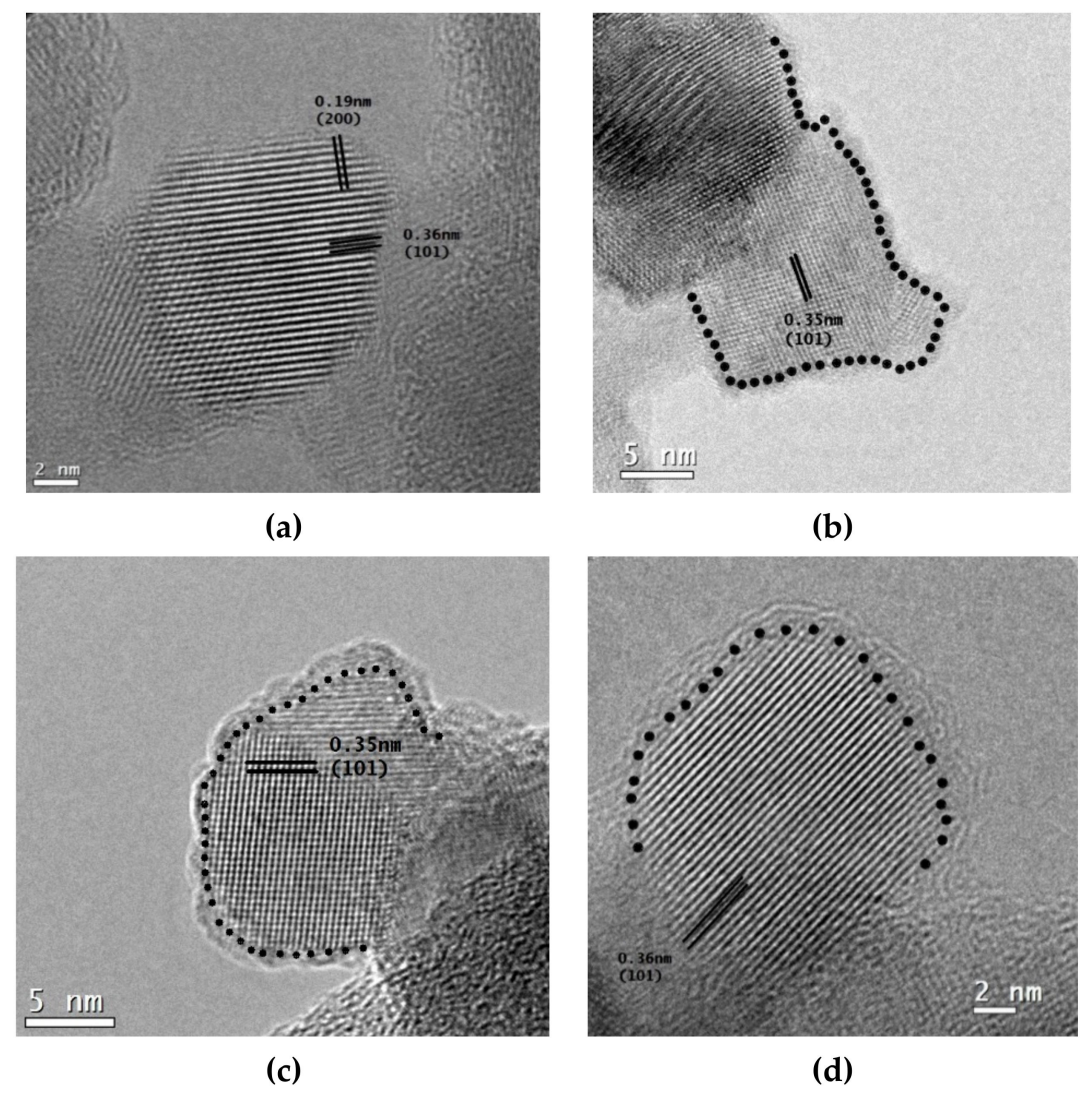
HRTEM images of TiO_2_ nanocrystals: (**a**) P25 TiO_2_ before reduction, (**b**) TiO_2_^red 30^ (1.2 nm of the amorphous layer), (**c**) TiO_2_^red 50^ (1.5 nm of the amorphous layer), and (**d**) TiO_2_^red 70^ (2.0 nm of the amorphous layer).

Micrographs of high-resolution transmission electron microscopy (HRTEM) of P25 TiO_2_ materials are presented in Figure 4, while the reduced forms of NF-TiO_2_, and NFP-TiO_2_ are included in the Supplementary Materials (Figure S1). The high crystalline order of the TiO_2_ nanoparticles with the characteristic lattice of anatase possessing indexed planes of (101) and (200) was observed in all samples. A thin amorphous layer was observed at the surface of the reduced TiO_2_ nanoparticles, indicative of surface modification. The measured thicknesses of the amorphous layers are tabulated in Table 1. The values of the amorphous layer and the characteristics of the crystalline order of the anatase suggest that we can modulate the amorphous layer by changing the duration of thermal treatment and consequently modulate the band gap energies into the visible light region. 

### 2.3. Energy Dispersive X-ray Fluorescence (EDXRF) Analysis

Our results demonstrated effective doping of TiO_2_ with nonmetals N, F, and P. Chemical composition as weight percentage for the synthesized catalyst NF-TiO_2_ was N (7.17%), O (42.46%), F (3.40%), and Ti (46.97%) and for the NFP-TiO_2_ the composition was determined as N (9.17%), O (37.28%), F (12.56%), P (7.35%), and Ti (33.65%). The XRF spectrum of doped samples NF-TiO_2_ and NFP-TiO_2_ are presented in Supplementary Materials (Figure S2). Degussa P25 has an average crystalline size of 21 nm with a specific surface area of 50 m^2^/g [54]. The particle size of 22.3 nm was calculated for the NFP samples processed at 600 °C, with a modest increase to 29.5 nm at 1000 °C and 40.7 nm 1100 °C reported [11]. 

### 2.4. Ultraviolet-visible (UV-Vis) and Raman Spectroscopy

The colors of the prepared TiO_2_, presented in Figure 2, indicate visible light absorption. The UV-visible diffuse reflectance spectra of the reduced TiO_2_ samples, provided in Supplementary Materials (Figure S3), were used to determine the specific absorption edges and band gap energies. The results demonstrated the band gap energies of doped samples shift to the visible light region compared to the unmodified P25 TiO_2_. A reduction of the band gap energy from 3.1 eV to 2.23 eV was observed for P25 TiO_2_ after treatment for approximately one hour. For NF-TiO_2_ particles, the band gap energy dropped by 0.8 eV to 1.30 eV. The NFP-TiO_2_ particles possess a band gap of 2.7 eV, decreasing to 2.35 eV upon reduction. The insertion of N atoms into TiO_2_ lattices is reported to create a new interband state above the valence band of TiO_2_ leading to the observed absorbance shift into the visible light region [55]. Since the absorbance edges of the prepared samples shifted into the visible light region, we assigned surface modification and production of an amorphous layer on the TiO_2_ particle results to changes in the band gap energy and visible light absorption properties. The band gap energies calculated by Kubelka–Munk plots are summarized in Table 2. 

**Figure 5:**
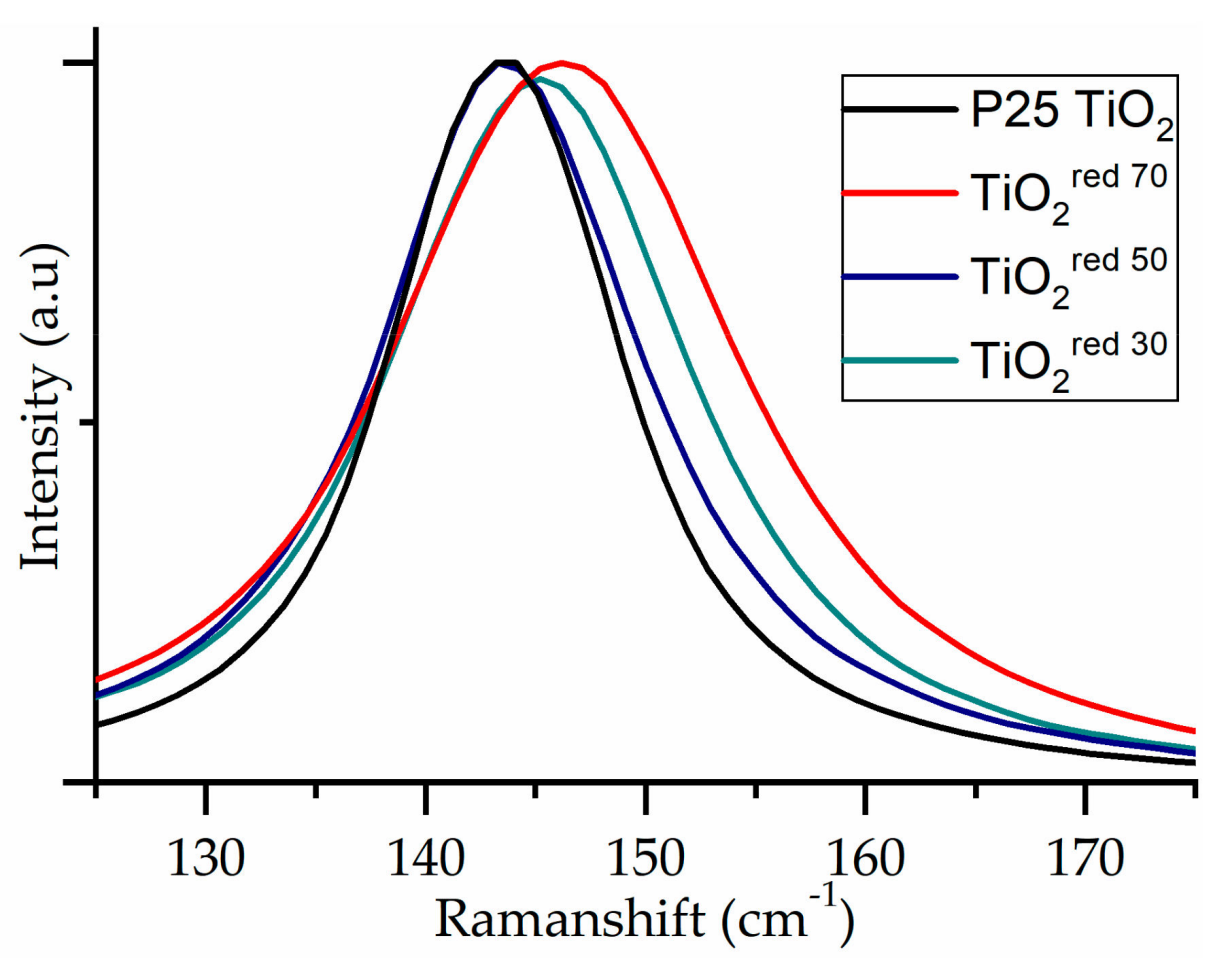
Raman spectrum of P25 TiO_2_ series showing shift toward the visible region as a function of reductive treatment time.

Raman spectra of the TiO_2_ materials exhibited the characteristic bands corresponding to Eg1, Bg1, Ag1, and Eg3 at 146, 396, 516, and 637 cm-1, respectively (data shown in Supplementary Materials Table S1), for the vibrational modes of the anatase phase of TiO_2_ [56]. Small shifts of the vibration mode were observed, as shown in Figure 5. The modest shift is assigned to the surface disorder as a result of the amorphous layer over the TiO_2_ nanoparticle (possible formation of oxygen vacancies or nonstoichiometry defects). Amorphous surface layers can also lead to peak broadening. Oxygen vacancy promotes the charge carrier separation and can potentially enhance the photocatalytic performance [41].

### 2.5. Photocatalytic Hydroxyl Radicals (•OH) Production

**Figure 6:**
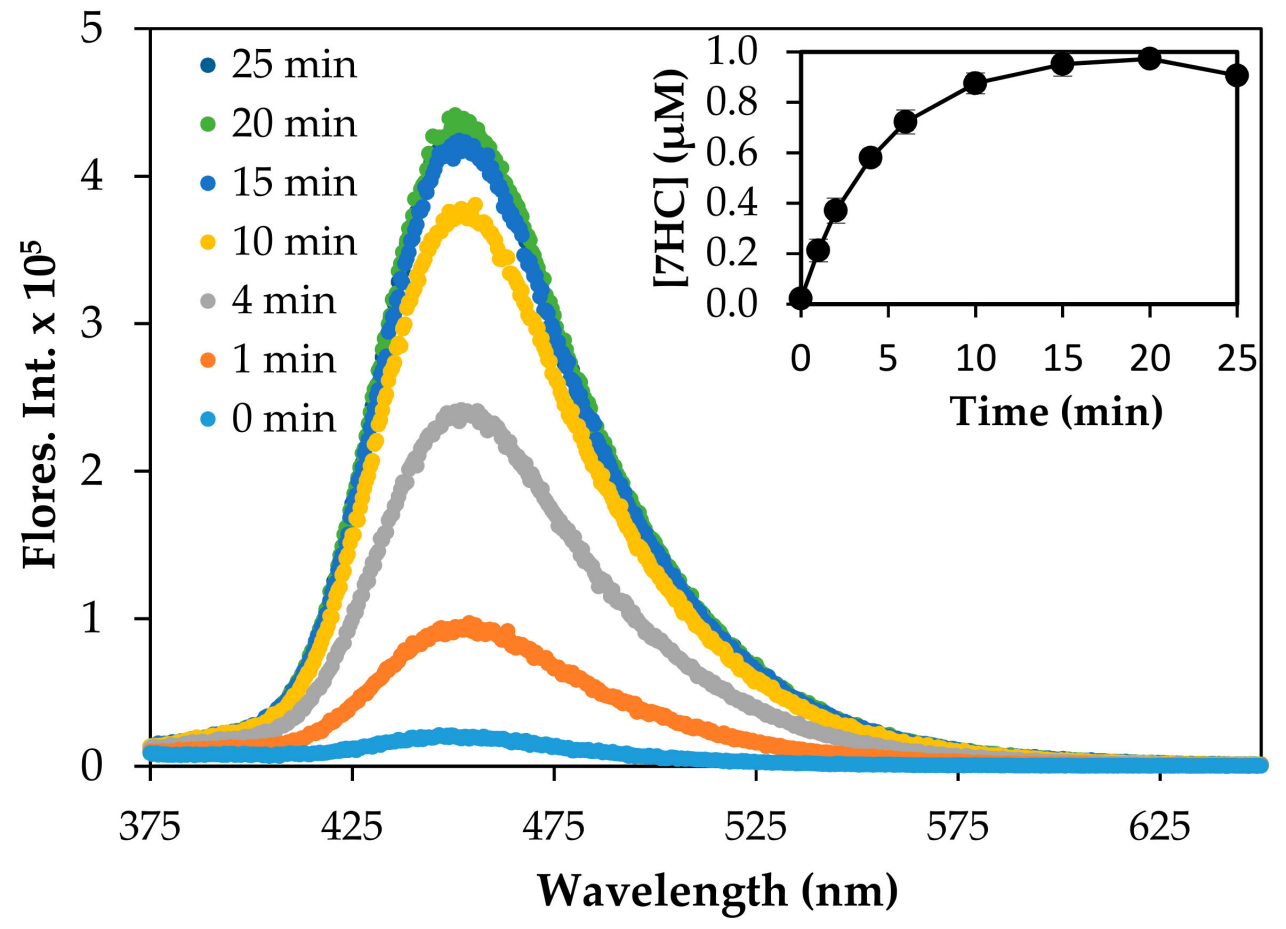
Change of fluorescence intensity observed at 455 nm during the irradiation of P25 TiO_2_ (0.10 g/L) suspended in a 125 µM COU aqueous solution. The plot of 7HC vs irradiation time is shown in the inset. The irradiation of P25 TiO_2_ suspension was carried out at 350 nm.

The production of the •OH adduct, 7HC, was monitored to assess the production of •OH by the reduced TiO_2_ materials at different irradiation wavelengths. Figure 6 shows the growth of fluorescence intensity at 455 nm of the hydroxyl radical trapping agent, 7HC, as a function of irradiation time under 350 UV light in the presence of P25 TiO_2_, as a reference for comparing different photocatalysts. Control experiments in the dark and in the absence of P25 TiO_2_ did not yield 7HC. The inset of Figure 6 shows the concentration of 7HC in P25 TiO_2_ aqueous suspension as a function of irradiation time. Triplicate experiments yielded reproducibility within 95%. The yield of 7HC increased in a linear fashion initially and reached a maximum at approximately 1.0 µM at 20 min of irradiation. Upon continued irradiation, the production of 7HC gradually decreased due to the likely depletion of coumarin, as well as the simultaneous formation and degradation of 7HC at higher concentrations. The formation of 7HC followed pseudo-zero-order kinetics in accordance with previous reports [46,57]. An apparent pseudo-zero-order rate constant (k_app_) of 0.16 µM min^-1^ (*R^2^* = 0.99) was determined under our experimental conditions. The observed rate constant was different from the rate constant of 0.023 µM min^-1^ reported by Nagarajan et al. [46], likely due to differences in experimental conditions including starting concentration, flux intensity, reactor design, etc. The concentration of 7HC (1.0 µM) produced at 20 min was equivalent to [•OH] = 16.4 µM, assuming trapping efficiency of 6.1% of total •OH [46,57]. 

### 2.6. Evaluation of the Photocatalytic Performance

**Figure 7:**
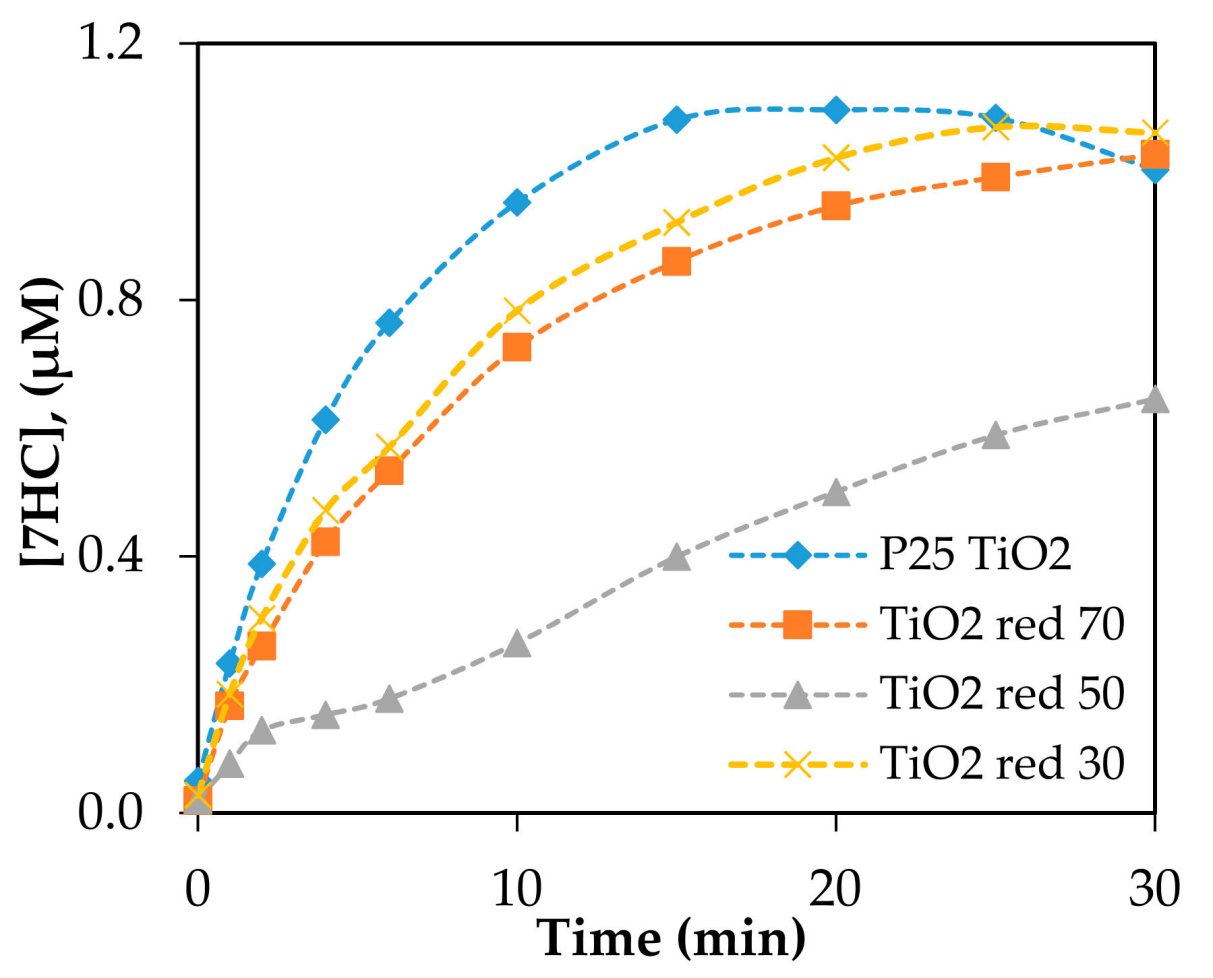
The production of 7HC by reduced P25 TiO_2_ photocatalysts irradiated at 350 nm. Catalyst loading was 0.10 g/L; [COU] = 125 µM.

The photocatalytic production of 7HC of the reduced TiO_2_ materials was carried out under the same conditions employed for the baseline using P25 TiO_2_ irradiation at λ = 350, 419, and 450 nm. Significant amounts of •OH, quantitated based on the formation of 7HC, were produced by reduced P25 TiO_2_ under UV (350 nm) irradiation shown in Figure 7. The formation of 7HC by reduced TiO_2_^red^ was slower than the reference P25 TiO_2_ and followed the order of TiO_2_^red 30^> TiO_2_^red 70^> TiO_2_^red 50^. Under 350 nm, the productions of hydroxyl radicals by reduced, doped NF-TiO_2_ and NFP-TiO_2_ were insignificant (data shown in Supplementary Materials Figure S4). No direct correlation was observed with the treatment time by NaBH_4_, •OH production, and the band gap energies.

**Figure 8:**
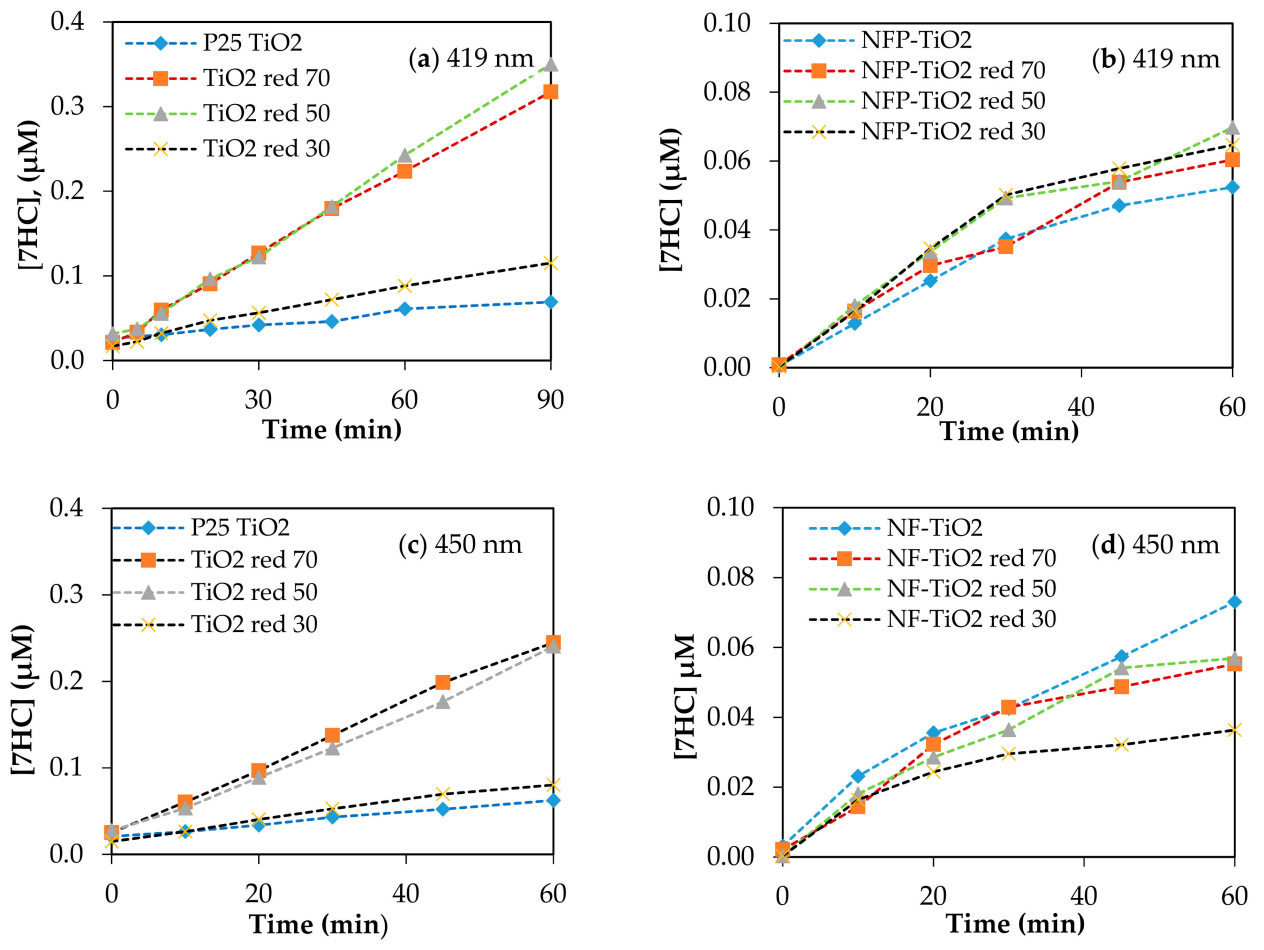
Production of 7HC using various TiO_2_ upon irradiation at visible light (**a**,**b**) 419 nm; (**c**,**d**) 450 nm. Catalyst loading was 0.10g/L; [COU] = 125 µM.

**Figure 9:**
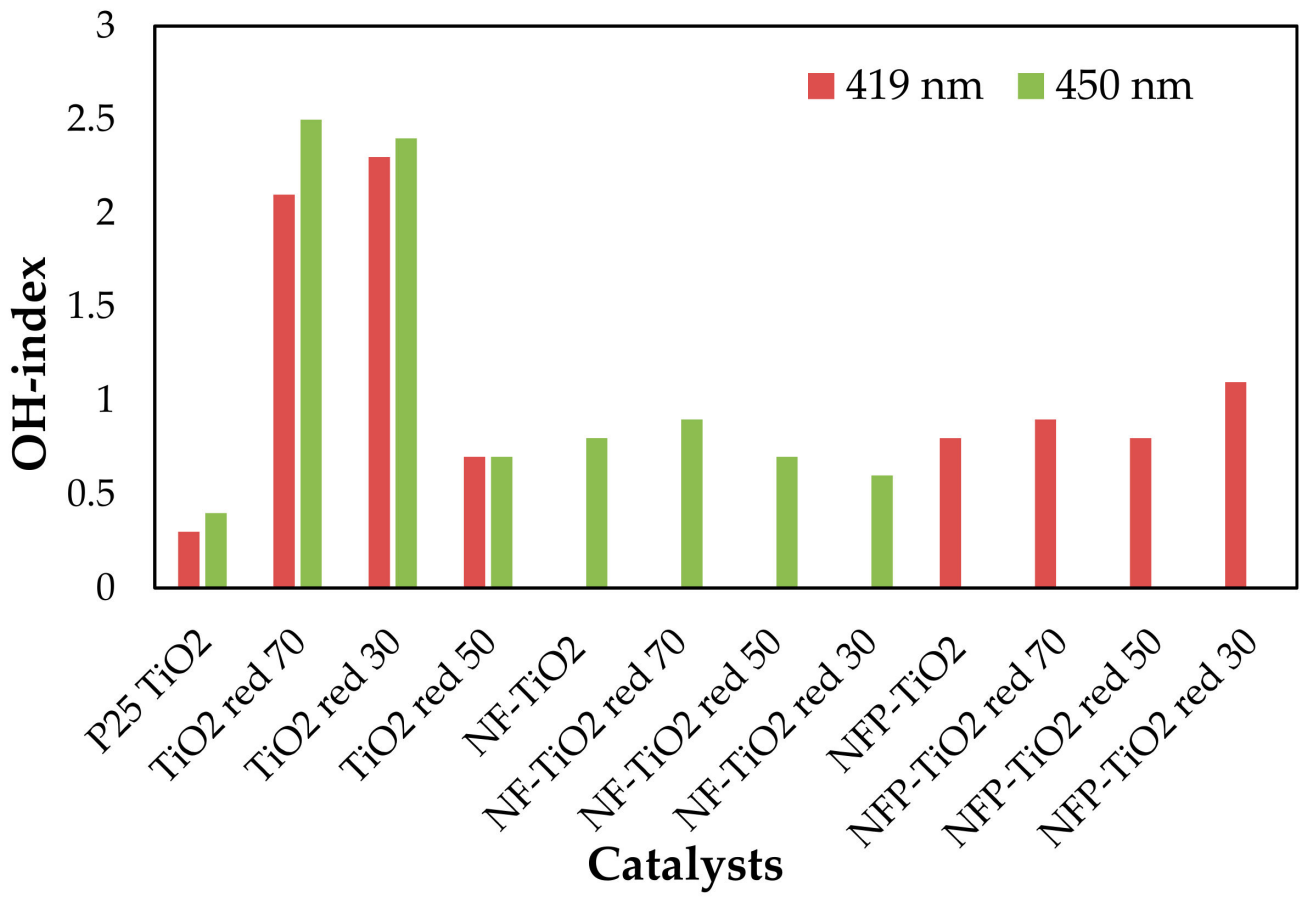
Relative comparison of OH-index of various TiO_2_-based photocatalysts at visible light (the data are reproducible with 5% of error based on some triplicate runs).

Visible light-activated photocatalysis has been reported to be effective for the degradation of organic pollutants [13,14,23,54]. The band gap of P25 TiO_2_ is 3.1 eV, and upon reduction with NaBH_4_, the band gap energy decreases to 2.23 eV, corresponding to light absorption wavelengths ~556 nm. Based on the calculated band gap for NF-TiO_2_ and NFP-TiO_2_ series, shown in Table 2, reduced NF-TiO_2_^red^ and NFP-TiO_2_^red^ may be active in the near IR region and at ~500 nm, respectively. The photocatalytic production of 7HC in aqueous suspension of the synthesized TiO_2_ materials under irradiation at 419 and 450 nm is presented in Figure 8. For quantitative estimation of their relative photocatalytic activity, we calculated OH-index for each of the prepared samples using Equation (6) based on the formation rate of 7HC correlated to the production of •OH for only the initial 10 min of reaction to avoid the possible degradation of 7HC. The calculated OH-indexes are summarized in Table 3. Though the formation rate of •OH depends on various factors, including phase structures, particle size, crystallinity, and surface defects, the composition is an important factor in the photolytic activity of TiO_2_ materials. A graphical comparison production of •OH at visible light is presented in Figure 9. 

In general, the larger the OH-index, the higher the photocatalytic activity. P25 TiO_2_ showed the highest OH-index for UV activation compared to the reduced TiO_2_ materials. P25 TiO_2_ is among the most active photocatalysts for the degradation of organic compounds initiated by •OH radicals under UV irradiation. However, under the visible light region, P25 TiO_2_ did not show appreciable photo activity. In the visible range, NaBH_4_ treated P25 TiO_2_, TiO_2_^red 70^, TiO_2_^red 50^, and TiO_2_^red 30^, showed enhanced photocatalytic activity compared to reference P25 TiO_2_. Though NFP codoped TiO_2_ exhibited limited photocatalytic activity under visible light, NaBH_4_ treatment did not make a difference in the performance. 

## 3. Materials and Methods 

### 3.1. Materials

P25 TiO_2_ donated by Degussa was employed as starting material. N and F codoped TiO_2_ (NF-TiO_2_) and N, F, and P codoped TiO_2_ (NFP-TiO_2_) were prepared by using titanium tetraisopropoxide (97.0%), isopropanol (99.0%), ammonium hexafluorophosphate (NFP) (≥95.0%), urea (N) (≥98.0%), trifluoroacetic acid (TFA) (99.0%), and phosphoric acid (PA) (85 wt.%) A detailed experimental procedure to prepare these samples is given in Ref [11]. NaBH_4_ obtained from Acros Organics was 98% pure and used without further purification. Coumarin was purchased from MP Biomedicals, LLC. 7-Hydroxycoumarin (7HC) was 99% pure and purchased from Acros Organics. The air used to purge samples was from Trigas of the highest available purity. Millipore water (18 MΩ∙cm) was used for the preparation of all aqueous solutions. 

### 3.2. Preparation of Reduced TiO_2_ Materials

In a typical procedure, 4.0 g of P25 TiO_2_ was mixed with 1.5 g of NaBH_4_ in an agate mortar and thoroughly ground with a pestle for 30 min at room temperature. The resulting mixture was transferred to a porcelain boat and placed in a tubular furnace. Gradient heat was applied at a rate of 10 °C min^-1^ from room temperature to 300 °C under an argon atmosphere and baked for an additional 30–70 min at 300 °C. The sample was removed from the furnace and allowed to naturally cool to room temperature. The resulting colored TiO_2_ was washed with deionized water and ethanol several times to eliminate unreacted NaBH_4_ and undesired byproducts. The washed sample was stored for characterization after drying at 70 °C for one hour. A series of reduced forms of NF-TiO_2_ (NF-TiO_2_^red^) and reduced forms of NFP-TiO_2_ (NFP-TiO_2_^red^) were prepared as a function of reduction time. 

### 3.3. Characterization of Reduced TiO_2_ Materials 

X-ray diffraction patterns were taken for the reduced TiO_2_ materials employing a Bruker Advance D8 X-ray diffractometer, with Cu Kα (λ = 1.54056) radiation with continuous scanning in the range of 10°< 2Θ <90°. Structure, surface morphology, and microstructure of prepared materials were characterized by high-resolution transmission electron microscope (HRTEM) imaging studies using FEI TITAN G2 80–300 operated at 300 kV. Samples were sonicated and placed onto a carbon-coated copper grid for scanning by microscope. The concentration of N, F, P, and Ti in doped TiO_2_ samples was determined using the energy dispersive X-ray fluorescence (EDXRF) analyses. The vibrational spectroscopy of hybrid nanostructure was identified using Thermo Scientific XDR Raman microscope employing 532 nm laser at 5 mW power. The UV-Visible absorption spectra of the various TiO_2_ materials were recorded at room temperature on a Varian Cary 100 Bio UV-Visible spectrophotometer in the wavelength range of 220–800 nm. The band gap of the prepared samples was calculated from Kubelka–Munk plots.

### 3.4. Photocatalytic Experiment

The illumination of the TiO_2_ suspension was carried out in a Rayonet Photochemical Reactor equipped with a cooling fan and up to 16 lamps in a merry-go-round arrangement (details of the reactor, model RPR-100, are available at The Southern New England Ultra Violet Company [58]. Three sets of lamps provide specific irradiation centered around 350, 419, or 450 nm. Eight lamps equally distributed within the reactor were employed in each experiment. A phosphor-coated black light of nearly 350 nm was used for UV irradiation and white tungsten bulbs of 419 and 450 nm for visible light irradiations. The intensity of light fluxes for 350 nm, 419 nm, and 450 nm was in 10% deviation according to manufacturer specifications. Pyrex glass reaction vessels with Teflon stoppers (L = 30 cm, D = 2.5 cm) were employed for all photocatalysis experiments. In a typical experiment, 100 mL of 125 µM COU aqueous solution was loaded with 10 mg of catalyst and sonicated in an ultrasonic cleaning bath for 10 min to produce a uniform of suspension. The mixture was gently purged with air for 10 min prior to irradiation. The aqueous suspension was magnetically stirred during irradiation in a photochemical reactor. The reaction vessel was removed from the reactor at desired time intervals, vigorously shaken for 30 s, and a 5.0 mL aliquot withdrawn from the reaction solution and immediately passed through a 0.45 µm PTFE filter. The filter was rinsed with another 5.0 ml of deionized water combined with filtrate to make the total volume of 10 ml for future analyses.

### 3.5. Measurement of the Formation of Hydroxyl Radicals (•OH) for Assessing the Photocatalytic Activities

The concentrations of •OH during irradiation of the aqueous TiO_2_ suspensions were measured from the concentration of highly fluorescent hydroxyl radical adduct (7HC) produced from the reaction of •OH with COU. Based on the established yield of 7HC from the COU + •OH reaction, the concentration of •OH production can be determined as a function of irradiation time. The optimal COU concentration ranges for •OH production are reported between 100–1000 µM [12]. Czili et al. observed maximum initial yields when the COU concentration was 100 µM [48]. While •OH generated during TiO_2_ photocatalysis is primarily formed at the surface, •OH_ads_ can diffuse from the TiO_2_ surface, leading to free •OH. Previous studies have shown free •OH, and •OH_ads_ occur at or near the surface and have similar reactivities. The trapping efficiency of •OH in TiO_2_ suspension reported by Zhang et al. is ~6.1% using 100 µM COU [57]. Newton and Milligan reported similar trapping efficiency of 4.7% [59]. The detection and quantification of 7HC were carried out by a Horiba FluoroMax Spectrofluorometer setting with excitation and emission wavelengths at 332 and 455 nm, respectively. The equipment was calibrated against a series of standard 7HC concentrations ranging from 0.1 to 3.0 µM (0.1, 0.5, 1.0, 1.5, 2.0, and 3.0 µM). The calibration plot, shown in Supplementary Materials (Figure S5) was obtained by plotting the fluorescence intensity measured at 455 nm as a function of 7HC concentration. The correlation coefficient of the calibration plot was ≥ 0.99. 

## 4. Conclusions

The visible light-activated reduced forms of TiO_2_ materials were prepared by a simple solid state thermo-physicochemical treatment using NaBH_4_. The materials were subsequently characterized by XRD, HRTEM, EDXRF, UV-Visible, and Raman spectrometry. Optical measurement suggested that physicochemical treatment can modulate the band gap energy-suitable properties for efficient photocatalytic activity. Coumarin was used as a fluorescent probe to trap and measure the •OH generated in catalyst suspension under UV and visible light irradiation. The photocatalytic performances of the synthesized reduced TiO_2_ materials were assessed by quantitative measurement of hydroxyl radical production under the irradiation of UV and visible light. Finally, OH-index of a photocatalyst using the formation rate of hydroxyl radicals was calculated to compare the relative photocatalytic activity of a photocatalyst. Though P25 TiO_2_ was found as the most active photocatalyst in the UV region, NaBH_4_-reduced forms of TiO_2_ were identified as significantly active photocatalysts under visible irradiation.

## Figures and Tables

**Table 1:** The prepared TiO_2_ materials as a function of reduction time with NaBH_4_ along with their thicknesses of the amorphous layer.

Series Name	Treatment Time with NaBH_4_ at 300 °C					
	30 min	Thickness(nm)	50 min	Thickness (nm)	70 min	Thickness (nm)
P25 TiO_2_	TiO_2_^red 30^	1.2	TiO_2_^red 50^	1.5	TiO_2_^red 70^	2.0
NF-TiO_2_	NF-TiO_2_^red 30^	1.3	NF-TiO_2_^red 50^	2.1	NF-TiO_2_^red 70^	6.0
NFP-TiO_2_	NFP-TiO_2_^red 30^	1.0	NFP-TiO_2_^red 50^	1.5	NFP-TiO_2_^red 70^	2.0

**Table 2:** Band gap of NaBH_4_ treated P25 TiO_2_, NF-TiO_2_, and NFP-TiO_2_ series and their corresponding photoexcited absorption wavelength.

Particles	ΔE (eV)	λ (nm)	Particles	ΔE (eV)	λ (nm)	Particles	ΔE (eV)	λ (nm)
P25 TiO_2_	3.10	400	NF-TiO_2_	2.10	590	NFP-TiO_2_	2.70	459
TiO_2_^red 30^	2.40	516	NF-TiO_2_^red 30^	1.43	867	NFP-TiO_2_^red 30^	2.51	494
TiO_2_^red 50^	2.30	540	NF-TiO_2_^red 50^	1.35	918	NFP-TiO_2_^red 50^	2.40	516
TiO_2_^red 70^	2.23	556	NF-TiO_2_^red 70^	1.30	953	NFP-TiO_2_^red 70^	2.35	527

**Table 3:** Calculated OH-index of prepared TiO_2_ materials.

Catalysts	OH-index		
	350 nm	419 nm	450 nm
P25 TiO_2_	100^A^	0.3	0.4
TiO_2_^red 70^	86	2.1	2.5
TiO_2_^red 30^	75	2.2	2.4
TiO_2_^red 50^	33	0.7	0.7
NF-TiO_2_	0.04	n/d	0.8
NF-TiO_2_^red 70^	0.03	n/d	0.9
NF-TiO_2_^red 50^	0.06	n/d	0.7
NF-TiO_2_^red 30^	0.05	n/d	0.6
NFP-TiO_2_	0.01	0.8	n/d
NFP-TiO_2_^red 70^	0.01	0.9	n/d
NFP-TiO_2_^red 50^	0.01	0.8	n/d
NFP-TiO_2_^red 30^	0.01	1.1	n/d

^A^ The data are reproducible with 5% of error based on some triplicate run. n/d represents no photocatalytic experiment of respective material for this condition was carried out.

## Supplementary Materials

The following are available online at https://www.mdpi.com/1420-3049/24/11/2147/s1, Figure S1a: HRTEM images of NF-TiO_2_. Figure S1b: HRTEM images of NFP-TiO_2_ nanocrystals, Figure S2: EDXRF spectrum of (a) NF-TiO_2_, and (b) NFP-TiO_2_, Figure S3: UV-Visible diffuse reflectance spectra of (a) P25 TiO_2_ series, (c) NF-TiO_2_ series, and (e) NFP-TiO_2_ series. The Kubelka-Munk plot for band energy calculation for (b) P25 TiO_2_ series, (d) NF-TiO_2_ series, and (f) NFP-TiO_2_ series, Figure S4: The production of 7HC at 350 nm by (a) reduced NF-TiO_2_ and (b) NFP-TiO_2_ photocatalyst, Figure S5: Spectrofluorometric calibration curve for measuring of 7HC. Table S1a: Raman band position of P25 TiO_2_ and reduced P25 TiO_2_ (TiO_2_^red^), Table S1b: Raman band position of NF-TiO_2_ and reduced NF-TiO_2_^red^, Table S1c: Raman band position of NFP- TiO_2_ and reduced NFP-TiO_2_^red^.
