# Supplementary material for: UV and Visible Light-Driven Production of Hydroxyl Radicals by Reduced Forms of N, F, and P Codoped Titanium Dioxide
Source: Molecules. 2019 Jun 6;24(11):2147. doi: 10.3390/molecules24112147 (PMC6600679; doi:10.3390/molecules24112147)
Supplement: Supplementary file 1 [file molecules-24-02147-s001.pdf]

*Supplementary Material*

# UV and Visible Light-Driven Production of Hydroxyl Radicals by Reduced Forms of N, F, and P Codoped Titanium Dioxide

**A. M. Abdullah [1], Miguel Á Gracia-Pinilla [2], Suresh C. Pillai [3] and Kevin O'Shea [1,*]**

[1] Department of Chemistry & Biochemistry, Florida International University, Miami, FL 33199, USA; am.abdullah@fiu.edu (A.M.A.); osheak@fiu.edu (K.O)

[2] Facultad de Ciencias Físico Matemáticas, Universidad Autónoma de Nuevo León, Av. Universidad s/n, Cd. Universitaria, San Nicolás de los Garza, Nuevo León 66455, México; miguel.graciapl@uanl.edu.mx

[3] Nanotechnology and Bio-engineering Research Group, Department of Environmental Science, Institute of Technology, Sligo, Ireland; pillai.suresh@itsligo.ie

* Correspondence: osheak@fiu.edu; Tel +1-305-348-3968

## HRTEM analysis

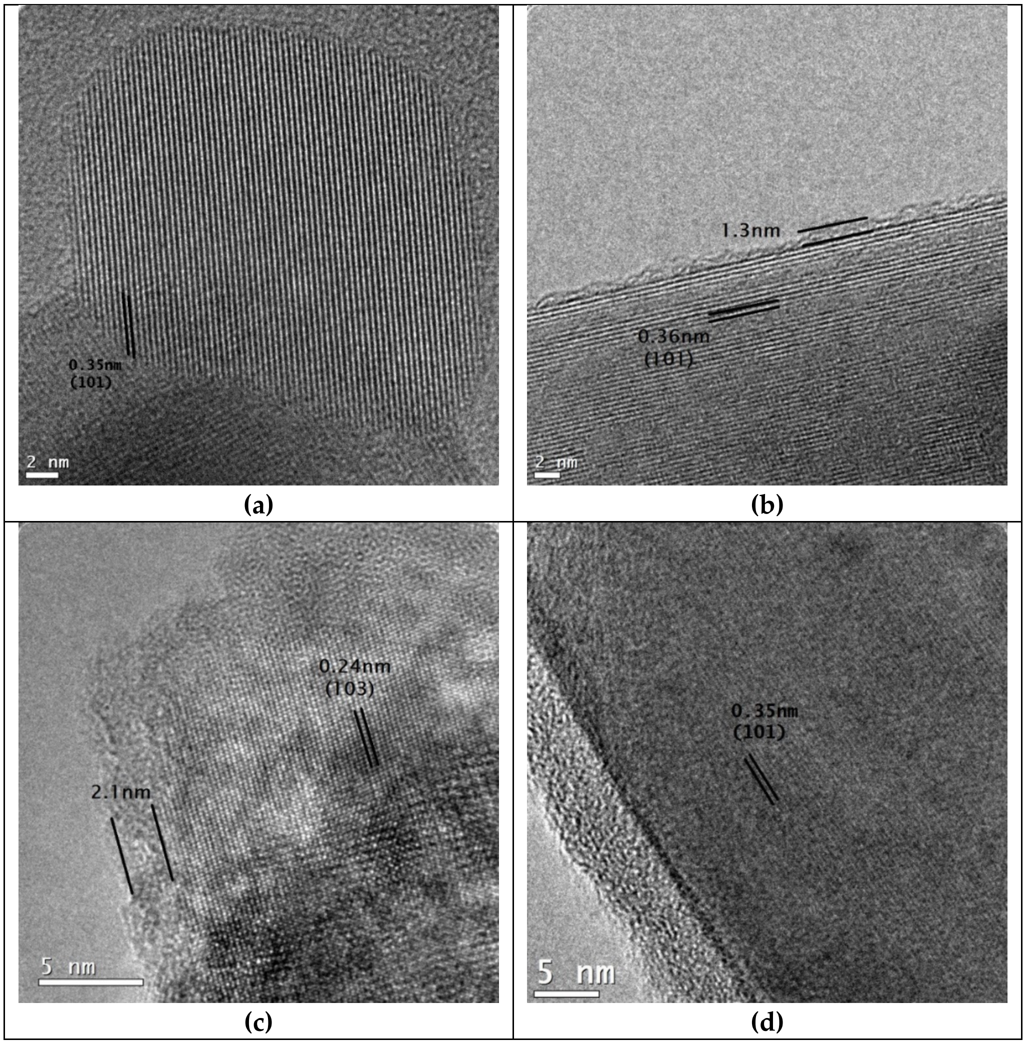


**Figure S1a**. HRTEM images of N, and F codoped $TiO_2$ nanocrystals (a) NF-$TiO_2$ before reduction, (b) NF-$TiO_2^{red\ 30}$ (1.3 nm of the amorphous layer), (c) NF-$TiO_2^{red\ 50}$ (2.1 nm of the amorphous layer) and (d) NF-$TiO_2^{red\ 70}$ (6.0 nm of the amorphous layer)

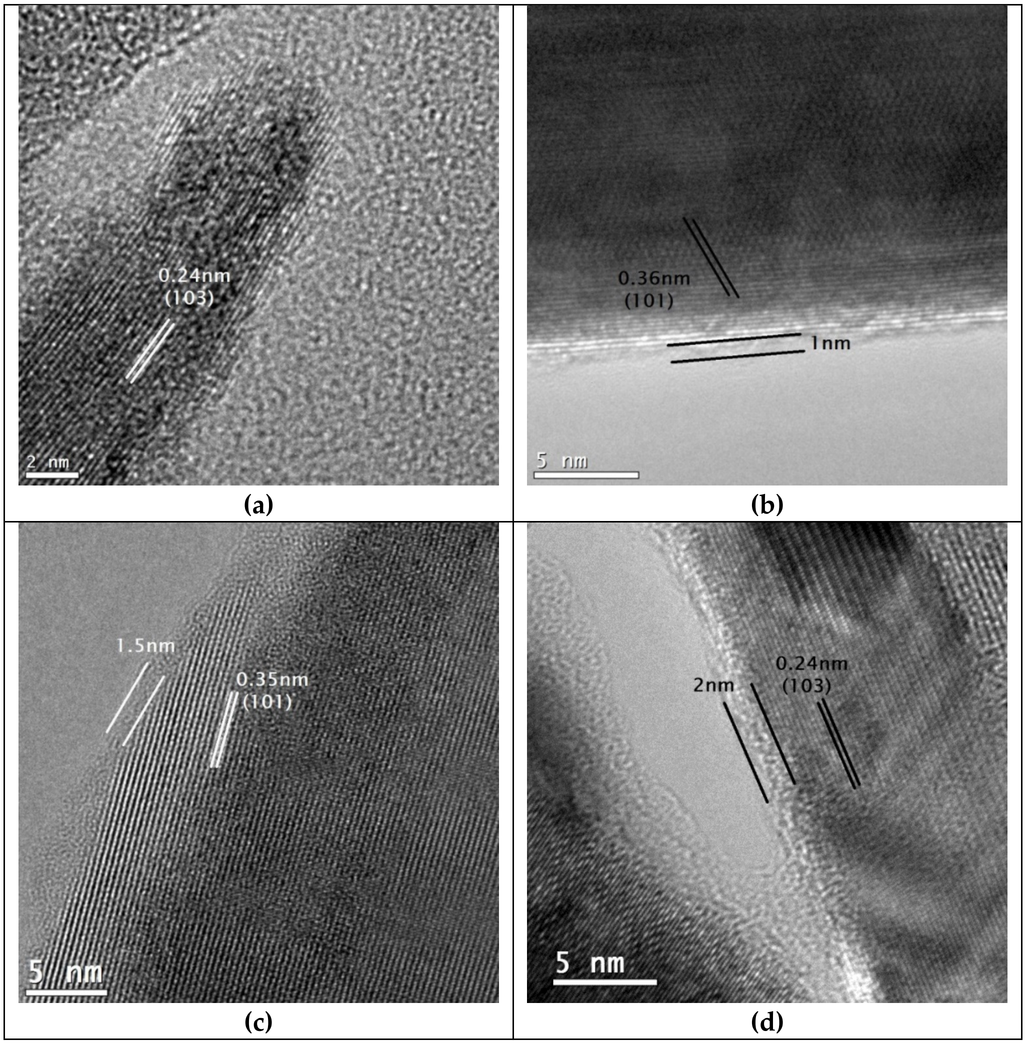


**Figure S1b.** HRTEM images of N, F, and P codoped $TiO_2$ nanocrystals (a) NFP-$TiO_2$ before reduction, (b) NFP-$TiO_2^{red\ 30}$ (1.0 nm of the amorphous layer), (c) NFP-$TiO_2^{red\ 50}$ (1.5 nm of the amorphous layer) and (d) NFP-$TiO_2^{red\ 70}$ (2.0 nm of the amorphous layer)

## EDXRF Analysis

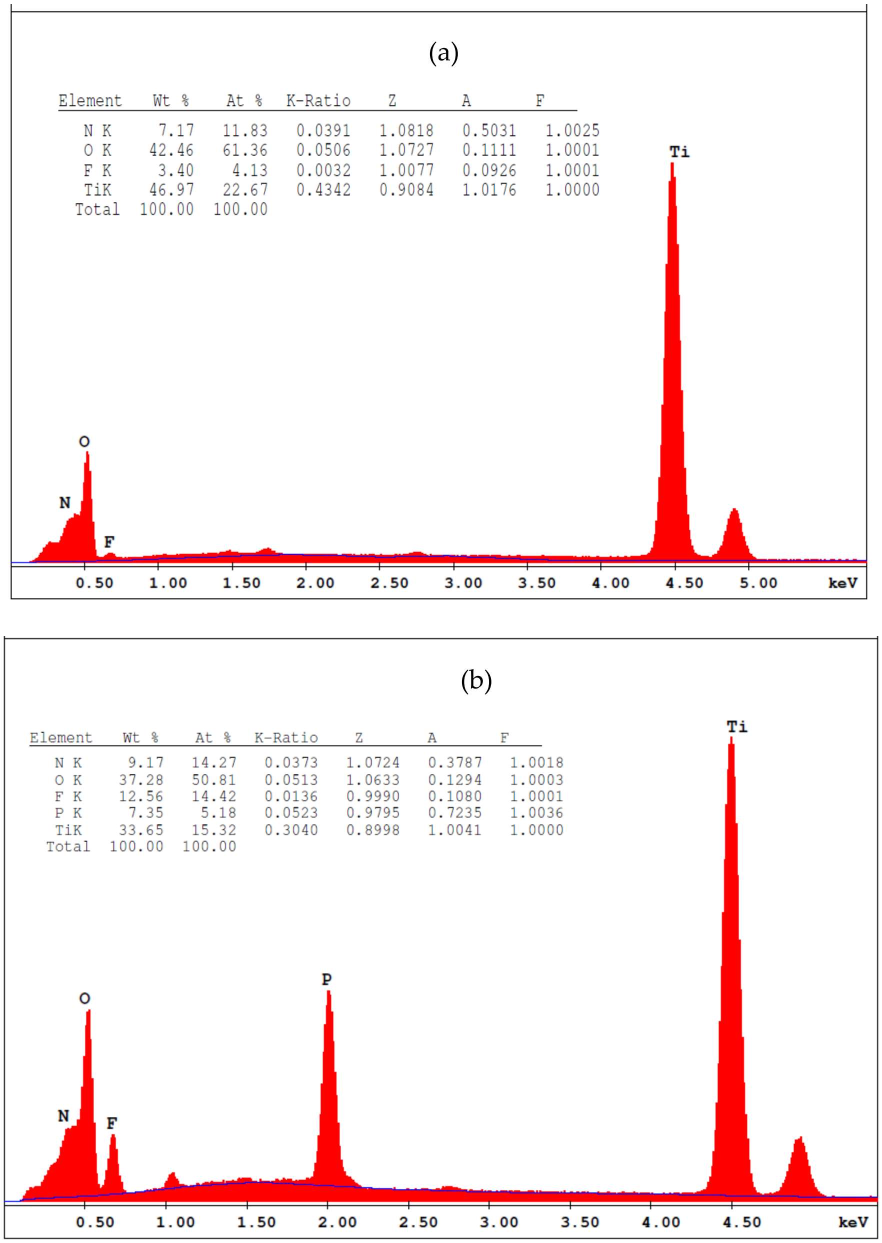


**Figure S2**. EDXRF spectrum of (a) N, and F codoped $TiO_2$ (NF-$TiO_2$) and (b) N, F, and P codoped $TiO_2$ (NFP-$TiO_2$)

## UV-Visible spectroscopy

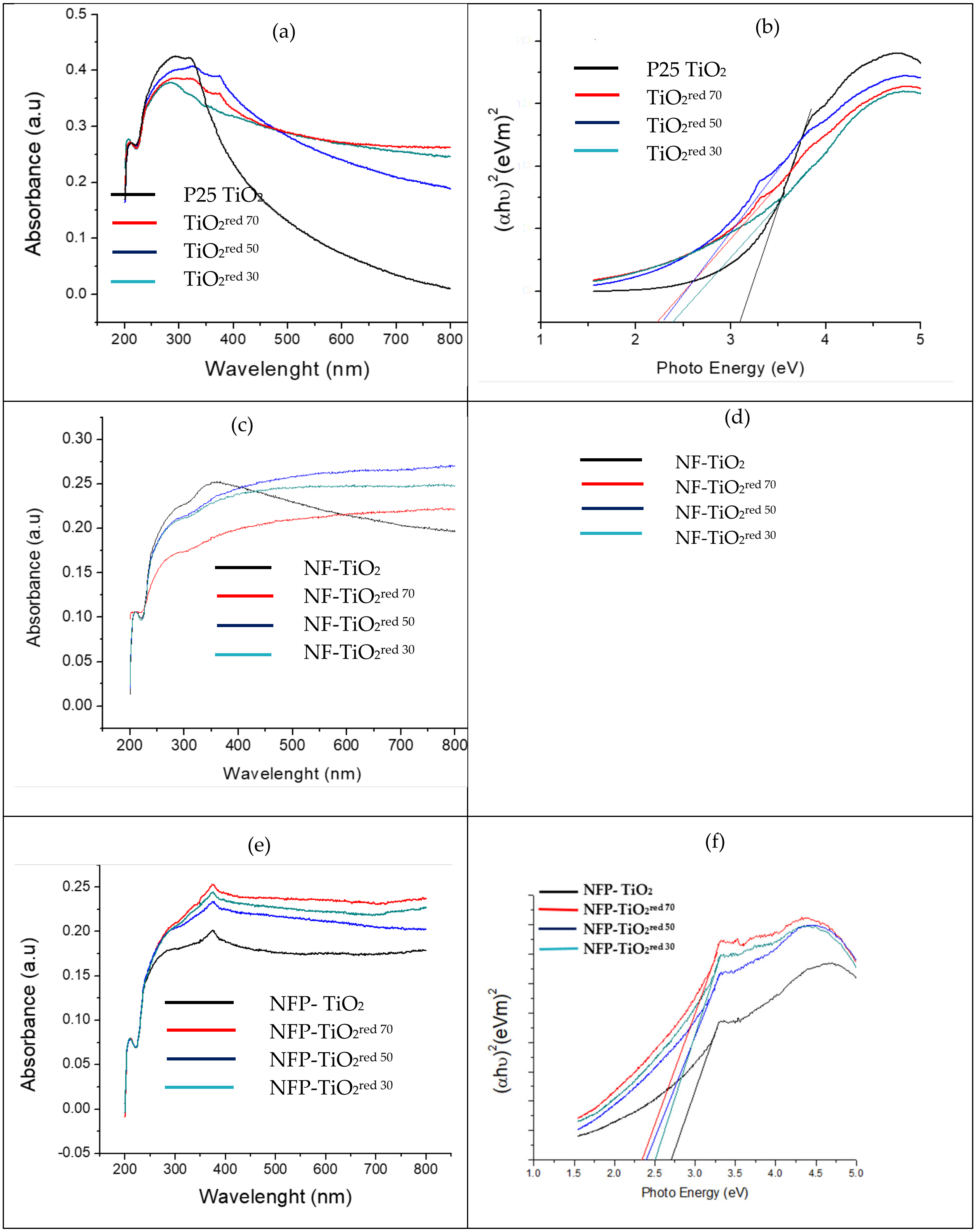

**Figure 3.** UV-Visible diffuse reflectance spectra of (a) P25 $TiO_2$ series, (c) NF-$TiO_2$ series, and (e) NFP-$TiO_2$ series. The Kubelka-Munk plot for band energy calculation for (b) P25 $TiO_2$ series, (d) NF-$TiO_2$ series, and (f) NFP-$TiO_2$ series.

**Raman Spectroscopy**

**Table S1a**. Raman band position of P25 $TiO_2$ and reduced P25 $TiO_2$ ($TiO_2^{red}$)

| **P25 $TiO_2$ ($cm^{-1}$)** | **$TiO_2^{red\,70}$ ($cm^{-1}$)** | **$TiO_2^{red\,50}$ ($cm^{-1}$)** | **$TiO_2^{red\,30}$ ($cm^{-1}$)** | **Band** |
|---|---|---|---|---|
| 143 | 146 | 143 | 145 | Eg1 |
| 396 | 394 | 395 | 397 | Bg1 |
| 516 | 511 | 511 | 521 | Ag1 |
| 637 | 634 | 634 | 636 | Eg3 |

**Table S1b.** Raman band position of NF-$TiO_2$ and reduced NF-$TiO_2^{red}$

| **NF-$TiO_2$ ($cm^{-1}$)** | **NF-$TiO_2^{red\,70}$ ($cm^{-1}$)** | **NF-$TiO_2^{red\,50}$ ($cm^{-1}$)** | **NF-$TiO_2^{red\,30}$ ($cm^{-1}$)** | **Band** |
|---|---|---|---|---|
| 142 | 144 | 145 | 144 | $Eg_1$ |
| 393 | 393 | 392 | 393 | $Bg_1$ |
| 512 | 511 | 512 | 511 | $Ag_1$ |
| 635 | 634 | 633 | 633 | $Eg_3$ |

**Table S1c.** Raman band position of NFP- $TiO_2$ and reduced NFP-$TiO_2^{red}$

| **NFP-$TiO_2$ (cm-$^{1}$)** | **NFP-$TiO_2^{red\,70}$ ($cm^{-1}$)** | **NFP-$TiO_2^{red\,50}$ ($cm^{-1}$)** | **NFP-$TiO_2^{red\,30}$ ($cm^{-1}$)** | **Band** |
|---|---|---|---|---|
| 143 | 148 | 147 | 149 | $Eg_1$ |
| 393 | 390 | 390 | 391 | $Bg_1$ |
| 511 | 506 | 509 | 507 | $Ag_1$ |
| 636 | 630 | 634 | 633 | $Eg_3$ |

**Production of hydroxyl radicals by reduced doped $TiO_2$**

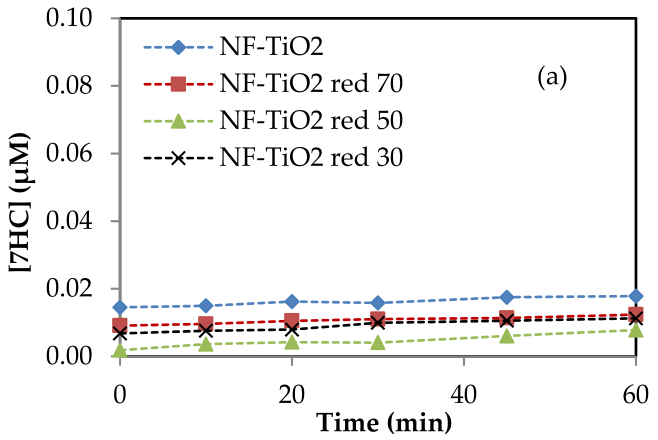


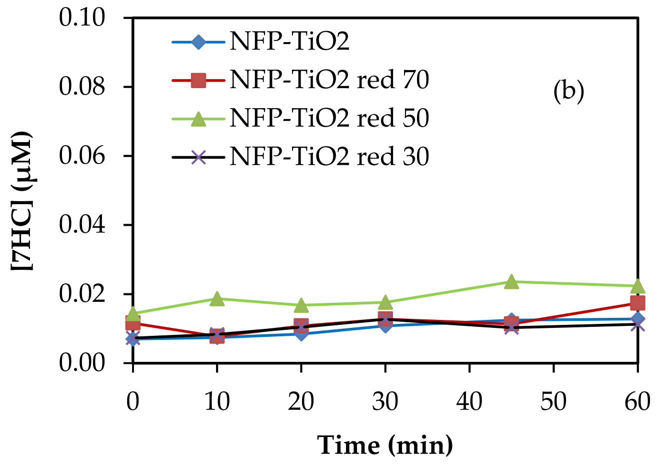


**Figure S4.** The production of 7HC at 350 nm by (a) reduced NF-$TiO_2$ and (b) NFP-$TiO_2$ photocatalyst

**Calibration curve for the measurement of 7-Hydroxycoumarin**

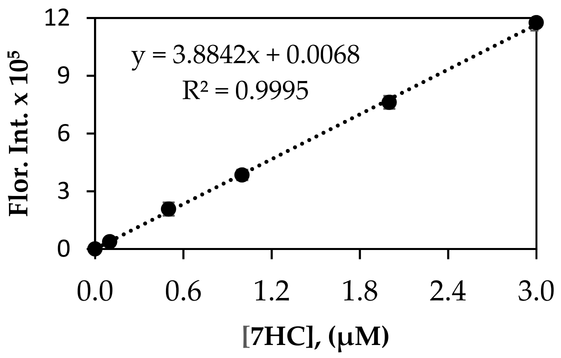


**Figure S5.** Spectrofluorometric calibration curve for measuring of 7HC. The data is reproducible within 5% standard deviation based on triplicate runs.
